# Weber's syndrome revealing a Percheron artery infarction: A case report

**DOI:** 10.1002/ccr3.7268

**Published:** 2023-04-23

**Authors:** Ahmadou Bamba Mbodji, Ibrahima Faye, Ndeye Rokhaya Diop, Moustapha Ndiaye

**Affiliations:** ^1^ Fann University Hospital Dakar Senegal; ^2^ Saint‐Louis Regional Hospital St.Louis Senegal

**Keywords:** ischemic stroke, neurology, Percheron artery, Weber syndrome

## Abstract

**Key Clinical Message:**

Weber's syndrome revealing a Percheron artery infarction is a rare clinical occurrence. Its diagnosis requires careful clinical examination and brain MRI, which is the gold standard for diagnosis. If this is not available, combined cerebral CT scan with a CT angiography of supra‐aortic arteries may be useful for the diagnosis.

**Abstract:**

Percheron's artery (PA) occlusion is an uncommon type of stroke involving paramedian thalamus and/or midbrain infarction. It accounts for 4%–18% of all thalamic infarcts and 0.1%–2% of all strokes. Its clinical manifestations are variable and its mode of presentation as Weber's syndrome is exceptional due to the unusual clinical presentation.

## 1 INTRODUCTION

The Percheron artery (PA) is an anatomical variant of the brain's posterior circulation arteries. Its occlusion will result in bilateral paramedian thalamic infarction with or without midbrain involvement.^1^ The clinical manifestations of ischemic stroke in this area can be summarized as a triad: altered consciousness, vertical gaze paralysis, and memory disorders.^2^ They are rarely associated with pyramidal signs or cranial nerve damage and the brain scan is usually unremarkable.^3^ This often leads to misdiagnosis in our low‐income countries where MRI (magnetic resonance imaging) is still not widely available. We report a case of Weber's syndrome secondary to Percheron artery occlusion.

## 2 CLINICAL CASE

We report the case of a 50‐year‐old hypertensive, for 5 years, under amlodipine 10 mg. She was admitted with consciousness disorders involving right hemiplegia. The exact time of the beginning of the symptoms was unknown. The patient was found unconscious.

**FIGURE 1:**
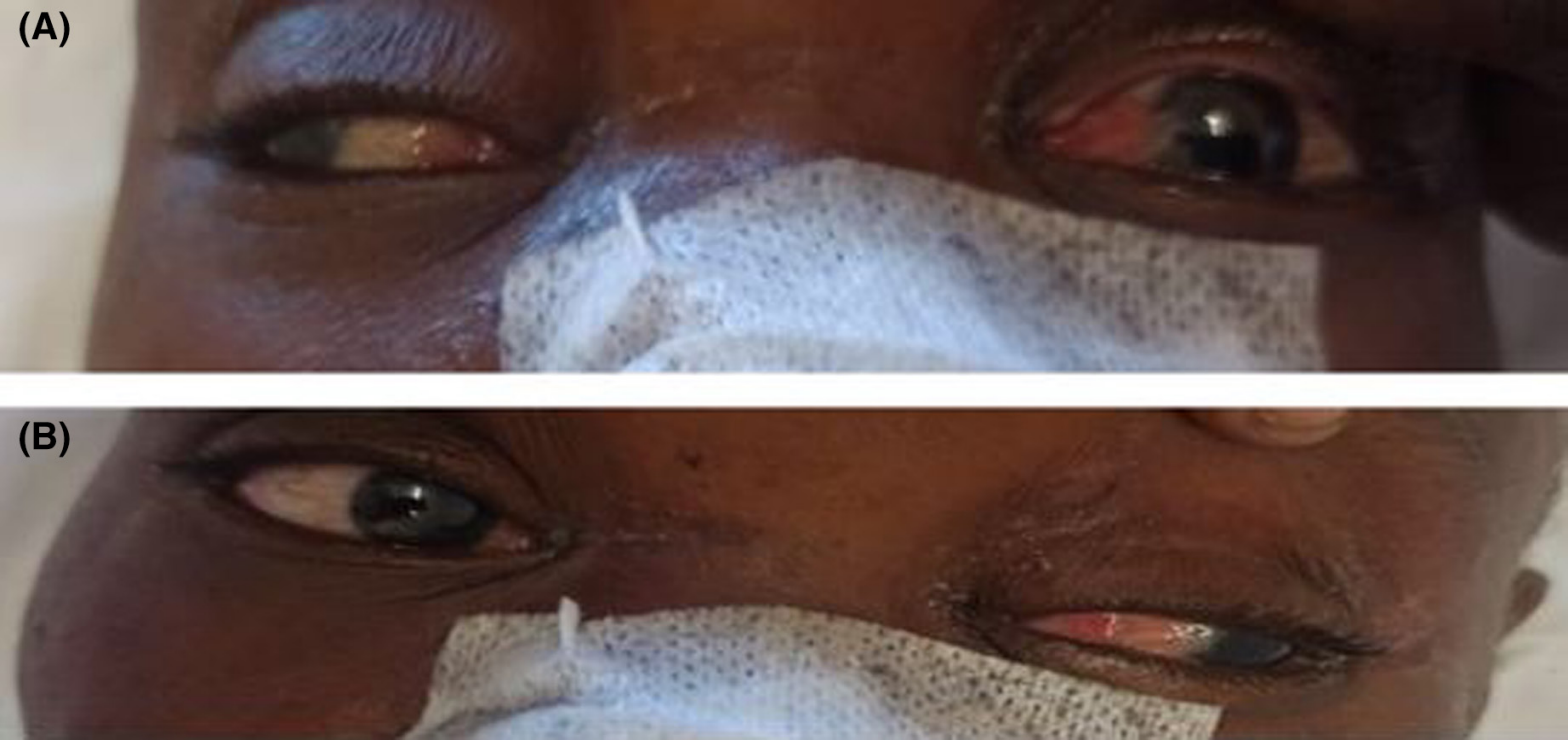
Abduction defect of the left eye.

On admission, the neurological examination showed a Glasgow scale of 9/15. The patient had drowsiness, fluctuating alertness with right hemiplegia, hypotonia, anesthesia, and areflexia associated with the Babinsky sign. The examination also revealed a lack of adduction of the left eye (during the conjugate deviation of the eye) associated with a dilated left pupil (Figure 1) and the initial NIHSS (National Institutes of Health Stroke Scale) score was 20. Blood pressure was 145/90 mmHg and casual capillary blood glucose was 1.15 g/L.

**FIGURE 2:**
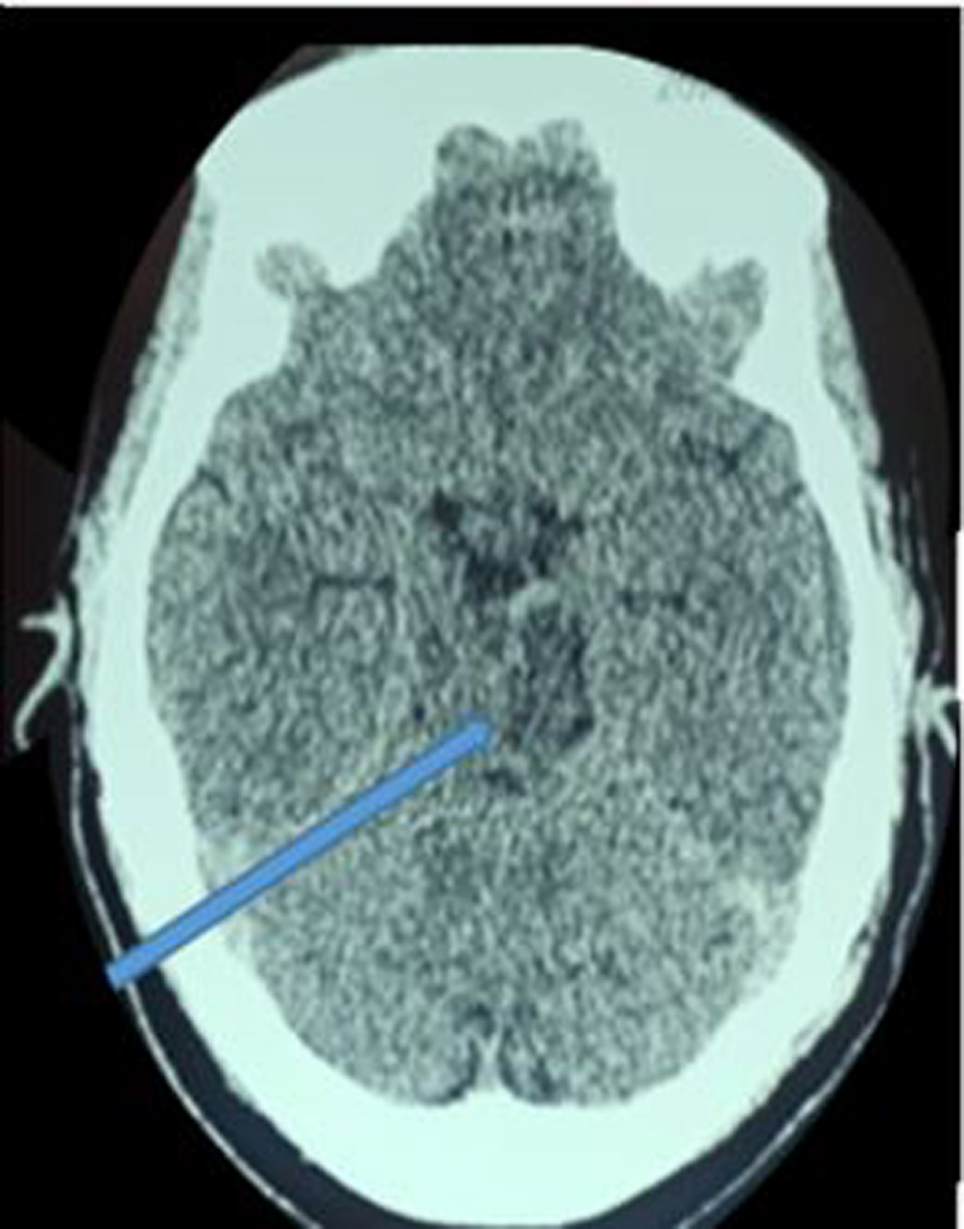
Ischemic lesion in the left mesencephalon.

**FIGURE 3:**
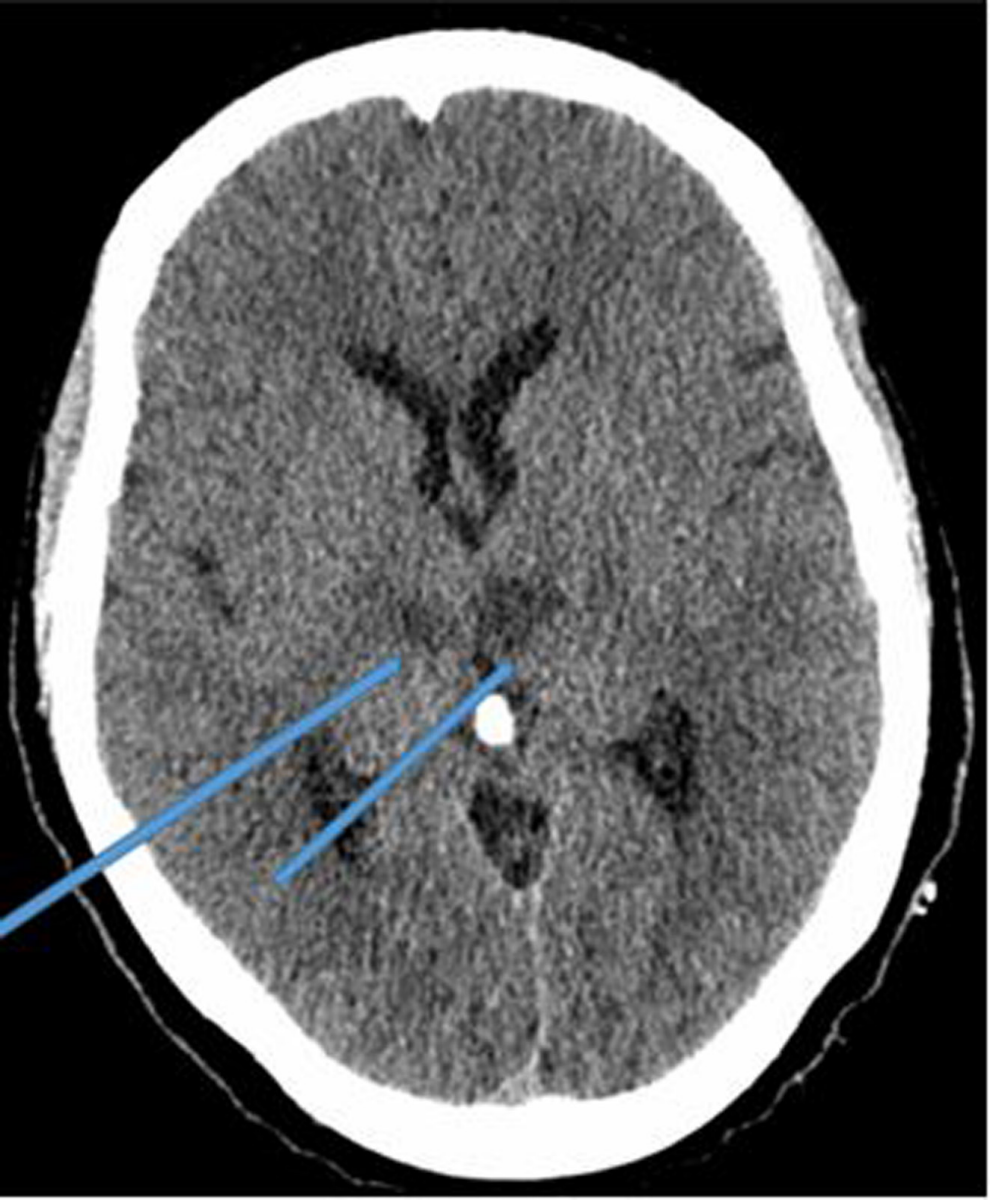
Ischemic lesions in the two thalami.

**FIGURE 4:**
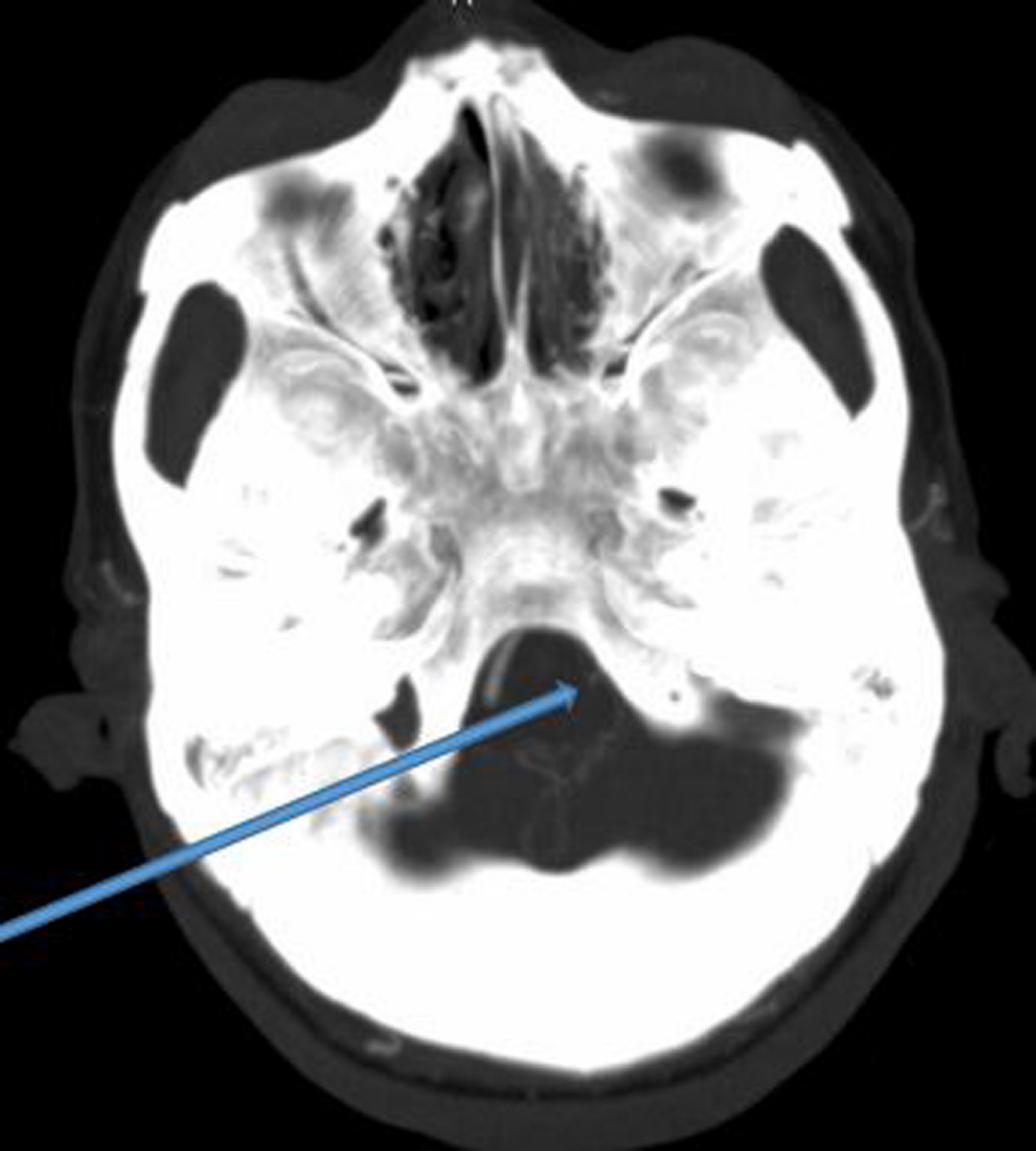
Occlusion of the left vertebral artery.

The brain CT showed a bilateral thalamic and mesencephalic infarction (Figures 2 and 3), the MRI was not performed because of its unavailability. CT angiogram of supra‐aortic arteries showed an occlusion of the V4 portion of the left vertebral artery (Figure 4). Selective cerebral angiography to make the diagnosis was not performed. The electrocardiogram and echocardiogram were normal but the 24‐h Holter electrocardiogram (EKG) did not show any rhythm disorder. The full blood count, urea, serum creatinine level, lipid profile, and blood glucose were normal but the thrombophilia assessment was not available. She was treated with 300 mg of aspirin and statins. The evolution was marked by the occurrence of an aspiration pneumopathy on Day 4 of hospitalization. This was successfully treated with amoxicillin/clavulanic acid. At 10 days of hospitalization, the consciousness was recovered. Until she was discharged, she underwent motor physiotherapy sessions associated with medical treatment. On discharge, she retained hemiplegia, motor aphasia, and third nerve damage. Her NIHSS score was 16. However, follow‐up was not performed because the patient was lost to follow‐up.

## 3 DISCUSSION

Cerebral infarcts affecting both paramedian thalami are unusual and may raise suspicion of occlusion of a single arterial trunk known as the Percheron artery.^4^, ^5^ This is the third anatomical arterial variant (Type IIb) that vascularized the thalami and/or the midbrain^3^ (see Table 1). These structures are responsible for the regulation of the sleep–wake cycle. Therefore, thalamic infarcts can cause paresthesia or numbness, speech and cognition disorders, memory impairment, and stupor.^6^ Our patient presented with a motor deficit associated with a stupor‐like consciousness disorder. This anatomical variant occurs in 4%–11% of the population and infarcts of this artery account for 4%–18% of all thalamic infarcts and 0.1%–2% of all strokes.^1^, ^7^, ^8^ Macedo et al. who had one of the most representative series found a frequency of 0.17%.^9^ The classic clinical presentation of Percheron artery infarction is a triad of altered consciousness, vertical gaze paralysis, and memory disorders.^2^ The association of these signs with Weber's syndrome in our patient constitutes one of the main particularities of our clinical case. Indeed, Weber's syndrome is part of the midbrain syndrome and is characterized by contralateral hemiplegia associated with an attack on the homolateral common oculomotor nerve. Its incidence is unknown and it rarely occurs in isolation.^10^, ^11^ Mesencephalic and Percheron artery infarction with Weber's syndrome is a rare and difficult clinical finding.^12^ In our patient, the common oculomotor nerve damage was not accompanied by pupillary damage. The most plausible hypothesis would be an absence of damage to the nuclei of the superior mesencephalon, responsible for pupillary innervation. Thus, a careful clinical examination allows diagnosis suspicion. Brain CT allowed to exclude the infarction involving the occipital lobe (or lobes), which could suggest basilar artery occlusion, but it did reveal symmetrical ischemic lesions in both thalami and anteromedial midbrain, which were consistent with clinical presentation.^6^, ^13^ Carotid and basilar Doppler ultrasounds were irrelevant. CT angiography was performed (arterial and venous phases), which revealed no signs of arterial or venous thrombosis. Although it did not show evidence of Percheron artery (PA) occlusion. It is not unusual as the Percheron artery is rarely visible on angio‐MRI or angio‐CT or conventional angiography. In our patient, the CT angiogram showed vertebral occlusion. Our patient did not benefit from intravenous thrombolysis because it was not available in our region. Finally, the treatment of Percheron artery infarction must be oriented according to the underlying pathological process. It can range from anti‐aggregation treatment to anticoagulation depending on the etiology.^14^ The long‐term outcome was not performed in our patient because he was lost to follow‐up. We could not determine if she presented persistent vigilance disorders or dementia as reported by Macedo et al. In his series of eight patients, he found hypersomnia in three patients.^9^

**TABLE 1:** Anatomical variations in the vascularity of the thalamus.

Anatomical description	
I	Normal anatomy of the paramedian artery: the left and right paramedian arteries arise from their respective posterior cerebral arteries
IIA variant	A variant of the paramedian artery anatomy: both paramedian arteries arise from the left or right posterior cerebral artery
IIB variant	Anatomy of Percheron artery: a branch of the right or left PCA supplies the two thalami
III	A variant of the paramedian artery anatomy: the two paramedian arteries originate from an arterial branch connecting the left and right posterior cerebral artery

## 4 CONCLUSION

The combination of consciousness disorders, oculomotor impairment, and hemiplegia should lead to the suspicion of brain stem damage and emergency imaging, in particular MRI, which is the gold standard. If the latter is not available, a brain CT associated with a CT angiogram of supra‐aortic trunks can be used for investigation. If the imaging reveals lesions in the thalami, Percheron artery infarction should be considered. Although Weber's syndrome associated with Percheron artery infarction is rare, the management is no different from other types of stroke and requires the same etiological approach. However, follow‐up is important to determine the evolution, which is variable depending on the patient.

## AUTHOR CONTRIBUTIONS

**Ahmadou Bamba Mbodji:** Writing – original draft; writing – review and editing. **Ibrahima Faye:** Writing – original draft; writing – review and editing. **Ndeye Rokhaya Diop:** Writing – original draft; writing – review and editing. **Moustapha Ndiaye:** Writing – original draft; writing – review and editing.

## FUNDING INFORMATION

The authors declared that they have no financial support to declare.

## CONFLICT OF INTEREST STATEMENT

The authors declared that they have no conflict of interest.

## CONSENT

Written informed consent was obtained from the patient's family to publish this report in accordance with the journal's patient consent policy.

## Data Availability

Data are available with the corresponding author upon request.
